# An approach for evaluating early and long term mother-to-child transmission of HIV (MTCT) in low and middle income countries: a South African experience

**DOI:** 10.1186/s12879-019-4336-1

**Published:** 2019-09-16

**Authors:** Debra J. Jackson, Thu-Ha Dinh, Carl J. Lombard, Gayle G. Sherman, Ameena E. Goga

**Affiliations:** 1 School of Public Health, University of the Western Cape, Cape Town, South Africa; 2 Health Section, UNICEF, New York, NY USA; 3 Centers for Disease Control and Prevention, Center for Global Health, Atlanta, GA USA; 4 Biostatistics Unit, Medical Research Council, Cape Town, South Africa; 5 School of Public Health and Family Medicine, University of Cape Town, Cape Town, South Africa; 6 Centre for HIV and STIs, National Institute for Communicable Diseases, National Health Laboratory Services, Johannesburg, South Africa; 7 Department of Paediatrics and Child Health, Faculty of Health Sciences, University of Witwatersrand, Johannesburg, South Africa; 8 Health Systems Research Unit, Medical Research Council, Pretoria, South Africa; 9 Department of Paediatrics, University of Pretoria, Pretoria, South Africa

**Keywords:** Study design, PMTCT effectiveness, Survey, South Africa

## Abstract

**Background:**

Eliminating mother-to-child transmission of HIV is a global public health target. Robust, feasible methodologies to measure population level impact of programmes to prevent mother-to-child transmission of HIV (PMTCT) are needed in high HIV prevalence settings. We present a summary of the protocol of the South African PMTCT Evaluation (SAPMTCTE) with its revision over three repeated rounds of the survey, 2010–2014.

**Methods:**

Three cross sectional surveys (2010, 2011–2012 and 2012–2013) were conducted in 580 primary health care immunisation service points randomly selected after stratified multistage probability proportional to size sampling. All infants aged 4–8 weeks receiving their six-week immunisation at a sampled facility on the day of the visit were eligible to participate. Trained research nurses conducted interviews and took infant dried blood spot (iDBS) samples for HIV enzyme immunoassay (EIA) and total nucleic acid polymerase chain reaction (PCR) testing. Interviews were conducted using mobile phones and iDBS were sent to the National Health Laboratory for testing. All findings were adjusted for study design, non-response, and weighted for number of South African live-birth in each study round. In 2012 a national closed cohort of these 4 to 8-week old infants testing EIA positive (HIV Exposed Infants) from the 2012–2013 cross-sectional survey was established to estimate longer-term PMTCT impact to 18 months. Follow-up analyses were to estimate weighted cumulative MTCT until 18 months, postnatal MTCT from 6 weeks until 18 months and a combined outcome of MTCT-or-death, using a competing risks model, with death as a competing risk. HIV-free survival was defined as a child surviving and HIV-negative up to 18 months or last visit seen. A weighted cumulative incidence analysis was conducted, adjusting for survey design effects.

**Discussion:**

In the absence of robust high-quality routine medical recording systems, in the context of a generalised HIV epidemic, national surveys can be used to monitor PMTCT effectiveness; however, monitoring long-term outcomes nationally is difficult due to poor retention in care.

## Background

In 2010 the WHO Global Elimination of Maternal to Child Transmission of HIV (MTCT) Initiative aimed to reduce new paediatric HIV infections by 90% by 2015 from the 2009 estimated baseline, and reduce the overall population-based HIV transmission rate through MTCT to < 5% (< 2% in the absence of breastfeeding) [1]. In 2011 the Global Plan to prevent MTCT and keep mothers alive was launched, identifying 21 Global Plan priority countries where 90% of the worlds’ HIV-positive pregnant women reside [2].

South Africa is defined as a high HIV prevalence country, with antenatal HIV prevalence of approximately 29.5% (95% confidence interval, 28.8–30.2), ranging from 16.9 to 37.4 across the nine provinces, and a commitment to reducing vertical HIV transmission [3–6]. In 2001, South Africa started implementing a programme to prevent HIV transmission from mother-to-child (PMTCT) at 18-pilot sites using a single dose Nevirapine (Sd-NVP) regimen for the mother during labour and to the baby within 72 h of delivery; modified obstetric practices; infant feeding counselling and the provision of free commercial infant formula to HIV-positive mothers who avoided breastfeeding [5]. The Sd-NVP PMTCT interventions were scaled up nationally in 2002 [6]. In 2008 the national antiretroviral regimens for pregnant women were changed to a dual therapy regimen defined as Azidothymidine (AZT) from 28 weeks with Sd-NVP at the outset of labour for pregnant women and Sd-NVP with AZT for baby [7].

In 2010, PMTCT interventions were further modified to include routine HIV testing and counselling for all pregnant women, and maternal triple antiretroviral therapy (ART) for pregnant women with CD4 cell count ≤350 cells/μl, or dual therapy from 14 weeks for those with ≤350 cells/μl, and postnatal infant prophylaxis for 6 weeks in non-breastfeeding infants or throughout the breastfeeding period (termed WHO Option A) [8]. In 2013 South Africa adopted WHO Option B (lifelong ART for all HIV positive pregnant women who require it for their own disease or through the end of breastfeeding for women with CD4 cell counts >350cells/mm^3^) [9]. In 2015 WHO Option B was extended to WHO Option B+, which recommended lifelong ART for all HIV positive pregnant and lactating women [10]. National guidelines recommended HIV PCR testing of all HIV-exposed infants at six-weeks, post breastfeeding cessations and 18 months post-delivery; however, with the adoption of PMTCT Option B+ HIV, PCR testing of infants was recommended at birth, 10 weeks, 6 weeks after breastfeeding cessation and 18 months [10].

Within 10 years of implementing the South African PMTCT program, national coverage increased to 95% of antenatal and maternity facilities by 2010. Between 2001 and 2010, data sources for PMTCT effectiveness assessments included: a) small scale evaluations in selected sites [11, 12], b) review of routine PCR positivity data from the National Health Laboratory Services (NHLS) [13] and c) review of routine data from the District Health Information system (DHIS) [14]. The small-scale surveys lacked a national footprint; the NHLS routine data potentially had selection bias in that it only reports on infants accessing testing, whilst the DHIS data lacked valid denominators to calculate transmission reliably. Consequently, there was a dire need to measure national PMTCT effectiveness by 2010. Therefore, the South African Medical Research Council, in collaboration with the South African National Department of Health, National Health Laboratory Services, CDC-PEPFAR, UNICEF and the University of the Western Cape, planned and implemented three national probability-based cross-sectional surveys in 2010, 2011 and 2012–13 to measure early national and provincial MTCT rates and a national cohort study in 2012–2014 to measure 18-month MTCT and HIV-free survival, to assess early and long term impact of the South African National PMTCT Programme.

This paper presents an overview of the South African PMTCT Evaluation (SAPMTCTE) protocol, covering both the cross-sectional and cohort components.

### Aims of the SAPMTCTE

We aimed to periodically measure the effectiveness and impact of the South African national PMTCT programme on mother-to-child transmission of HIV (MTCT), with surveys planned in 2010, 2011–12 and 2012–13. In 2012–13, we added additional primary objectives, which were to measure the cumulative MTCT from 6-weeks post-partum through 18 months post-delivery and HIV-free survival among a national cohort of infants born to HIV-positive mothers.

Secondary objectives of the surveys were to: a) estimate coverage of key PMTCT interventions and services (e.g. HIV testing, CD4 cell count testing, infant ARV prophylaxis, counselling on infant feeding); b) estimate the association between MTCT rate and ARV regimen, maternal background characteristics including CD4 cell count, maternal health care services and maternal and infant health status; c) measure uptake of HIV care-and-treatment referral services for HIV-infected pregnant women, infant post-test counselling and HIV-infected children who are diagnosed at primary health care setting; d) measure the impact of maternal ART or maternal/infant antiretroviral (ARV) prophylaxis on growth in the cohort of HIV exposed uninfected (HEU) infants up to 18 months; and e) identify potential factors associated with loss to follow up from the HEU cohort.

## Methods/design

### Study design and sampling frame

Cross-sectional national probability-based primary health care facility-based surveys, using an HIV biomedical marker (HIV antibody testing) to determine infant HIV exposure, and a virological test to measure perinatal HIV transmission amongst biomarker positive (HIV exposed) infants (HEI) were conducted at 4–8 weeks post-delivery from June–December 2010, August 2011–March 2012 and October 2012–May 2013 [15, 16].

The sampling frame for the primary sampling units (PSU) were public (government-funded) Primary Health Care (PHC) clinics and Community Health Centres (CHC) reportedly administering six-week immunisations (DPT1 or Pentaxim1 or the hexavalent vaccine), as documented in the South African National District Health Information System (DHIS). While other public facilities do administer immunizations (hospitals, mobile clinics), these PHC clinics and CHC are the primary locations for routine primary child health services. Data from the 2007 DHIS was used to obtain this sampling frame of eligible facilities and included the number of DTP1 administrations done in each facility for that year.

The sampling design was stratified by the nine provinces. Eligible facilities were stratified on the number of annual immunizations reported with three strata based on reported annual DTP1 administration: < 130, 130–300 and > 300 immunizations per annum. The stratum of the small facilities (< 130 immunizations per annum) consisted of 951 facilities performing 67,514 immunizations per annum which represented 7.0% of the total immunizations done in 2007 in South Africa. For fieldwork cost-efficiency, a strategic decision was taken to exclude this stratum from the formal sample. The National Department of Health expressed an interest in the efficiency of the PMTCT in facilities with high prevalence and high birth rates. To accommodate this request, facilities with > 300 immunizations per annum in the sampling frame were also stratified in two strata (≥29% or < 29%) based on the 2008 HIV national antenatal prevalence of 29%, using the district HIV prevalence in which they were based.

**Fig. 1:**
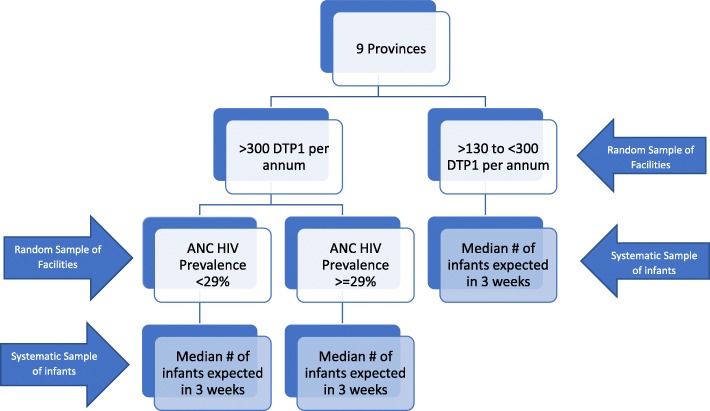
Sampling Frame

A two-stage stratified sampling was used. In the first stage, facilities (primary sampling units - PSUs) were randomly sampled proportional to size (PPS) within each of the 27 (9 provinces X 3 facility size/HIV prevalence) stratum (see Fig. 1). The method operated under the with-replacement-type selection [17]. At the second stage, a fixed number of infants per facility was systematically sampled. The fixed number was the median number of infants expected within the sampling window (3 weeks) across the population of facilities within the stratum as determined from the detailed information of the sampling frame above. The fixed number of infants sampled in each facility within a stratum ensured a self-weighting sample. A sampling window of 3 weeks was used to realize the required sample. Five hundred eighty facilities were sampled (approximately 21–22 per strata). More detail on sampling methods have been published by Goga, et al. 2014 & 2016 and Dinh, et al. 2015 [15, 16, 18].

From the 2012–13 survey, HEU infants were invited to participate in a closed, prospective observational 18-month follow-up study. Establishing and following-up the cohort entailed recruitment into the follow-up study at the time of the six-week interview, documenting detailed participant contact information (including address) in a protected web-based data base that only the allocated data collector had access to, and allowing home-based data collection and an inconvenience allowance of approximately 10 USD. HEU were followed up at 3, 6, 9, 12, 15 and 18 months to determine maternal and child access to care and MTCT. HIV infected children were followed up once after HIV positive diagnosis to determine access to HIV treatment. If the infant had not yet accessed care, appropriate referrals were made.

### Study population

The cross-sectional survey was conducted among all mother/legal caregiver-infant pairs (97% were biological mother) who presented at their local primary health care facility for their infant’s six-week immunisation (1st DTP dose) visit, regardless of their PMTCT status or HIV exposure. South Africa reports > 95% coverage of 6 week immunisation (1^st^DTP dose) [19], making these clinics an ideal catchment point for young infants. Infants accessing selected public primary health care clinics or community health centres were eligible for the cross-sectional surveys if they were: a) receiving their six-week immunization on the day of data collection, and b) 4–8 weeks old, c) did not need emergency medical care, and d) their mother/legal caregiver consented to participate in the survey. Infants were enrolled into follow-up if: a) their mother reported as being HIV positive and/or their HIV antibody test was positive, and b) they consented to follow-up. Infants remained eligible for on-going follow-up until 18 months if their PCR tests remained negative (i.e. HIV exposed uninfected infants). As noted above, if the infant PCR was positive at 6 weeks or became positive at any follow-up visit they received one additional follow-up visit to assess access to paediatric treatment, with appropriate referrals if infants had not accessed HIV care.

### Sample size

To determine the sample size for each province, HIV prevalence was calculated based on the provincial antenatal survey prevalence and coverage of PMTCT ARV prophylaxis. Estimates of transmission rates for Sd-NVP and no treatment were taken from Rollins [12] while the transmission rate for dual therapy came from a provincial survey from KwaZulu Natal province (unpublished data, 2009).

To balance samples across the nine provinces, absolute precision specified varied from 1 to 2%, with relative precisions of 22 to 60%. In general, provinces with a higher prevalence will have a lower (better) relative precision. These specifications resulted in better equity in sample size between provinces. Using this approach, the largest sample in a province was 1800 (Gauteng) and the smallest was 700 (Northern Cape) with a total sample size of 12,200 across all provinces (Table 1).

**Table 1:**
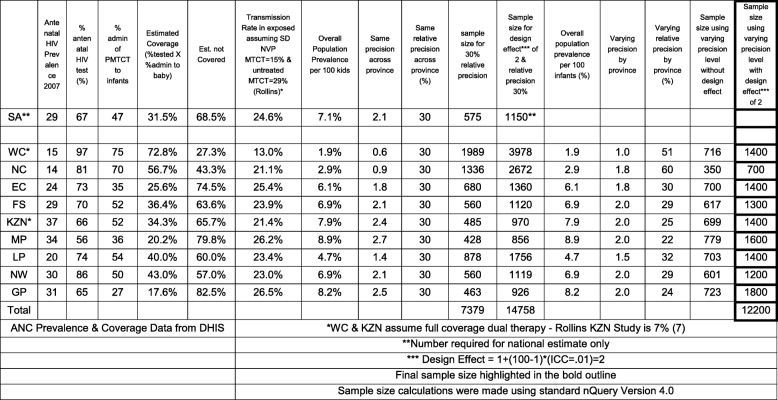
Sample size calculation for the cross-sectional surveys

Table 2 illustrates the sample size calculation for valid national estimates of 18 month MTCT and HIV-free survival. The red box in Table 2 highlights the target sample size selected for 18-month MTCT, and the green box highlights the target sample size selected for HIV-free survival estimates. These calculations demonstrated that assuming a design effect of 2, 18-month MTCT of 5% (3.5–6.5%) and 10% (7–13%) HIV infection or death, 1620 infants were needed to estimate MTCT and 768 infants are needed to estimate HIV infection or death with the indicated precision. Assuming 30% loss to follow-up, 2314 infants needed to be enrolled into the follow-up study to estimate both MTCT and HIV-free survival.

**Table 2:**
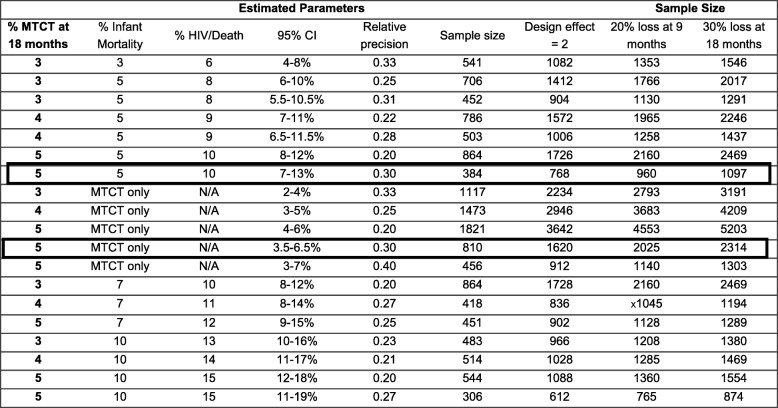
Sample size calculation for follow-up component

Footnote: The bolded boxes highlight the sample sizes selected to estimate HIV infection or death (green box) or 18-month MTCT only (red box), with 30% precision, a design effect of 2 and assuming 20 or 30% loss to follow-up. These show that assuming a design effect of 2, 18-month MTCT of 5% (3.5–6.5%) and 10% (7–13%) MTCT or death, 1620 infants are needed to estimate MTCT and 768 infants are needed to estimate HIV or death. Assuming 30% loss to follow-up, 2314 infants need to be enrolled to estimate MTCT and HIV-free survival. Sample size calculations were made using standard nQuery Version 4.0

### Data collection and study tools

Trained nurse data collectors recruited mothers/caregivers from the PHC/CHC waiting room during immunisation days. Data collectors introduced themselves and the study verbally and in written form using a standardised information sheet. A screening questionnaire was administered to determine eligibility and then full informed consent was completed for eligible infants (see below). If an eligible mother/legal caregiver-infant pair agreed to be interviewed, the interview was conducted in a private location.

Data were gathered using a questionnaire adapted from several validated tools [11, 12, 20]. The questionnaire included information on maternal age, parity, socio-economic status, antenatal care, HIV testing, maternal HIV status, PMTCT care during pregnancy and delivery, infant feeding counseling, birth information, infant feeding practices, infant weight; immunisations, postnatal visits and illness since birth. Legal (non-maternal) caregivers were administered a shorter version of the questionnaire that excluded antenatal care and PMTCT programme information.

Trained research nurses also collected infant heel prick dried blood spot samples during infant immunization visits, from all consented infants, regardless of reported maternal HIV status or ARV exposure. The blood testing replaced the routine testing for HIV PCR as part of the PMTCT programme during the period of the study as it was felt to be inappropriate to bleed a child twice during the same clinic visit. Trained research nurses worked from immunization clinics to facilitate standardization of procedure and data quality control. They offered routine HIV testing to all infants attending the clinic for immunization, thus preventing the testing of a potentially biased sample of infants: routine PMTCT EID clinics only test known HIV-exposed infants. Additionally, we found that maternal request for early infant diagnosis of HIV infection (EID) during the routine immunization services were low (47%) [21]. The mothers/caregivers were informed that the infant testing would also act as a biomedical marker for HIV status of the mother and that she may need to have further follow-up including HIV counselling and testing (HCT).

For the cohort study, the same trained research nurses used contact details obtained at cohort enrollment to arrange to meet the mother/legal caregiver-infant pairs during routine follow-up for all HEU at the health facility at 3, 6, 9, 12, 15 and 18 months. Interviews were completed and iDBS were taken for HIV PCR at 3, 6, 9, 12 and 15 months as the national guidelines did not recommend routine blood testing during these visits. During the 18 month visit the routine in-clinic HIV rapid test was used to document 18-month infant HIV status. The iDBS were sent to a centralized accredited laboratory (National Institute for Communicable Diseases, a division of the National Health Laboratory services) for HIV enzyme immunoassay (EIA) testing, followed by HIV total nucleic acid polymerase chain reaction (TNA PCR) testing on EIA positive samples. All EIA positive samples were serially tested for HIV infection with a second HIV EIA test, and 10% of negative samples were re-tested using a second test. Discordant first and second EIA test results underwent additional testing using Western Blot. Details about laboratory procedures have been published previously [15, 16].

Questionnaire data were entered in a mobile phone platform and transferred electronically to a central server for data management and data analysis. This is described in detail in another paper in this supplement [Ref pending].

### Ethics

The Ethics Committee of the South African Medical Research Council (SAMRC), provincial Ethics Committees, and the Centers for Disease Control and Prevention, Atlanta, approved the study protocol. All eligible mothers/primary caregivers (14+ years of age) were taken through a written informed consent process. Pregnant adolescents age 14–17 are considered emancipated minors in South Africa and were eligible for study participation. The process was conducted in the language of the participant and information sheets were translated into all South African official 11 languages. Consent forms were completed in duplicate, and one copy was given to the participant and confidentiality discussed and the other sent to the SAMRC offices. At the end of the interview, if the mother reported being HIV-positive and hence eligible for the follow-up study, a second consent process was conducted, followed by the completion of a second consent form. A participant contact information form was completed for all participants who agreed to follow-up and this was stored in a password protected database and could only be accessed by the data collector allocated to that participant. All mothers and infants were referred into care (routine maternal HIV testing or CD4 cell count testing or ART) as appropriate using referral cards which stipulated the exact, most convenient and appropriate routine health care setting that could to be accessed for further care. These sites were determined following a situational assessment conducted prior to the surveys. Determining appropriate referral sites for each study clinic and establishing referral protocols for HIV-positive mothers and infants is an important ethical consideration for any PMTCT evaluation. If this information is not already established, then a situational assessment to map referral sites may be needed prior to PMTCT study implementation [21].

### Data analysis and sample weighting

Sample weights were calculated for the cross-sectional surveys to adjust for differential sampling design across provinces and the sample realization. To achieve this, the data from provinces were weighted by using the proportional distribution of live births per province recorded in 2008. The realisation weights were done at various levels (district, provincial) depending on the sample size realisation within strata. All findings were adjusted for study design, non-response, and weighted for number of South African live-birth in each study round.

For the cohort study, longitudinal data were weighted for ‘no consent’ and ‘loss to follow-up’ amongst HIV exposed uninfected infants eligible for follow-up. Follow-up analyses were to estimate weighted cumulative MTCT until 18 months, postnatal MTCT from 6 weeks until 18 months and a combined outcome of MTCT-or-death, using a competing risks model, with death as a competing risk. HIV-free survival was defined as a child surviving and HIV-negative up to 18 months or last visit seen. A weighted cumulative incidence analysis was conducted, adjusting for survey design effects.

Specific details of each analysis will vary depending on analysis objective, but will generally include both descriptive (rates, means, 95% confidence intervals) and analytic analyses (chi-square, ANOVA or logistic regression, with 95% confidence intervals around adjusted estimates).

## Discussion

Several global working groups have published pros and cons of various methodologies which could be considered for evaluating PMTCT impact [22, 23]. The first publication in 2012 [22] reviewed in detail a variety of potential methods for measuring impact of PMTCT programmes, including: modelling, facility-based survey and follow-up, cohort/follow-up data, population-based household surveys, and analysis of early infant diagnosis and child HIV testing data. The 2015 guidance focuses more on monitoring and evaluation frameworks, including impact evaluations [23]. The 2015 guidance highlights the advantages of routine cohort data for measuring long term PMTCT programme impact if the following conditions are met: unique identifiers, HEI HIV PCR results available, with > 80% retention in care and analysis of final infant outcome data. These two guidance documents can assist potential evaluators to make decisions about what methodology or mixture of methodologies to employ to answer defined evaluation questions [22, 23].

### Main aims of the SAPMTCTE

The SAPMTCTE protocol outlined in this paper aimed to measure MTCT, HIV-free survival and PMTCT programme outcomes at national and provincial levels, used a facility and probability based survey method with infants attending public child health services, and was conducted as an independent evaluation, outside of routine systems using mobile electronic data collection.

### Limitations of cross-sectional and cohort studies to measure the impact of PMTCT programmes

Limitations of the cross-sectional SAPMTCTE must be recognized as it serves as a baseline for the follow-up component. The data were primary facility-based using infants presenting for immunization and routine care-services for HIV-exposed children. Infants who did not come for immunization or had already died by 6 weeks of age or attended private hospital/clinic for care were not included in the sample suggesting a possible selection bias over-estimating PMTCT effectiveness.

Mothers may have not accurately reported or documented HIV status or ARV adherence practices for a variety of reasons, such as fear of stigma and disclosure. Confidentiality was assured and discussed as part of the informed consent process and a private place was secured for the conduct of interviews to reduce this potential limitation. A strength of the study was that maternal HIV infection was based on both self-report of HIV status and presence of HIV antibodies in infant ELISA test. This allows for detection of infant HIV exposure even when mothers reported HIV negative, due to hesitance to report HIV status or due to incident infection during pregnancy.

The main limitation of the prospective follow-up component, which was conducted within a routine care system, was no consent for participation in the follow-up component and loss-to-follow up amongst those who agreed to participate. However, we factored in loss-to-follow up rate into our sample size calculation, and weighted the data for no consent and loss to follow up. We were not able to predict the directions of this bias. Furthermore, we relied on self-reported data on infant feeding and antiretroviral use (maternal and infant) and did not collect drug names, dosages or blood levels to verify reported information. We did not collect viral load data to relate measured MTCT with maternal viral suppression postnatally.

### Advantages of cross-sectional and cohort studies to measure the impact of PMTCT programmes

The strength of this approach was that this was a first probability based national survey of MTCT which included all provinces and all districts in South Africa facilitating national and provincial estimates and tracking of MTCT and PMTCT programme coverage.

This study provides a robust, feasible methodology to measure population level impact of programmes to prevent mother-to-child transmission of HIV (PMTCT) in a high HIV prevalence setting. An added advantage over other studies is that this design provides programme coverage and client behavior data. These are not provided by aggregated programme data or laboratory data.

### Policy and programme implications

The results of surveys such as the SAPMTCTE have guided policy and programs for PMTCT to reach an HIV-free generation. All results were discussed during district and provincial-level PMTCT stock-taking and EMTCT workshops, and guided planning. Results were also presented at PMTCT Technical working group meetings and influences policy changes around repeat HIV testing during pregnancy and introducing a birth and 10 week HIV test. These methods provide both impact and granular programmatic data which can be used to improve national and provincial PMTCT programming, and to target programming to achieve equitable results for all.

## Data Availability

Further details on study design and methodology can be found in the final study reports found at: http://www.mrc.ac.za/reports/evaluation-effectiveness-prevention-mother-child-transmission-hiv-pmtct-programme-south?bc=285 [Last accessed 7 Aug 2019].

## Abbreviations

AZT: Azidothymidine
CHC: Community Health Centres
DHIS: District Health Information system
EIA: Enzyme-linked immunosorbent assay
EID: Early infant diagnosis
HCT: HIV counselling and testing
HEI: HIV exposed infant
HEU: HIV-exposed uninfected
HIV: Human immunodeficiency virus
iDBS: Infant dried blood spot
MTCT: Mother-to-child transmission of HIV
NHLS: National health laboratory services
PCR: Polymerase chain reaction
PHC: Primary Health Care
PMTCT: Prevention of mother-to-child transmission of HIV
PSU: Primary sampling units
SAPMTCTE: South African prevention of mother-to-child transmission of HIV programme evaluation
Sd-NVP: Single dose Nevirapine
TNA PCR: Total nucleic acid polymerase chain reaction
WHO: World Health Organization
